# Tracing and Tracking of Food as a basis for “smart agrifood”

**Published:** 2014

**Authors:** Hallier Bernd

**Affiliations:** * EHI Retail Institute, Germany

**Abstract**

**Economy:**

- worldwide accessibility for food/diminishing quantitative discrepancy between rich and poor countries

- providing a nutritional optimum per capita

- decreasing waste of food

**Food Security:**

- tracing/tracking of animals and products

- developing a good agricultural practice

- building responsibility for the total supply chain

**Sustainability:**

- meeting technical standards like CO 2 decrease/not living on the expense of other generations

- developing eco-ethics (2) : for consumption and mental behaviour/promoting eating culture 

to care for human issues like social balance (nationally/internationally)

**Keywords:** tracing, tracking, smart agrifood, EHI

**Holistic approach for future harmony**

**Figure:**
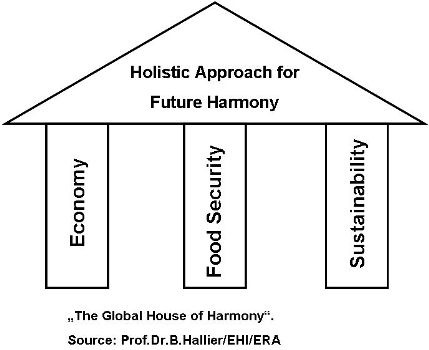


**The Empowerment of Retail by marketing-tools**

**Figure:**
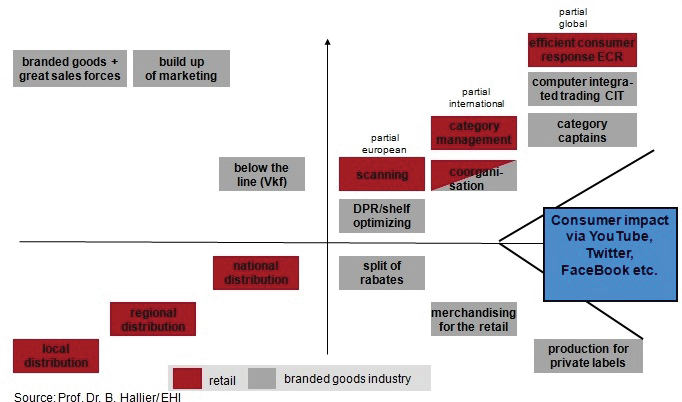


EHI has own researchers – and more than 2000 experts from the retail industry in its workshops

EHI Retail Institute has three important subsidiaries:

- 50% GS1 Germany (since 1974)

- 50% Orgainvent (Tracing/Tracking)

- 100% GlobalGAP (Good Agricultural Practice)

**Case Study 1**

All started with the European Article Numbering System EAN – initiated by Albert Heijn (Ahold)

Normal version of the 13-digit EAN

**Figure:**
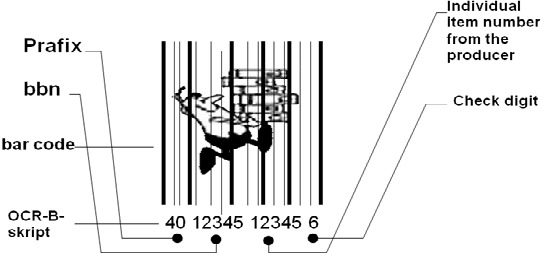


**New Technologies**

The next step had been RFID (Radio Frequency Identification) and WLAN (Wireless Local Area Network)

**Parameters with an influence on RFID**

**Figure:**
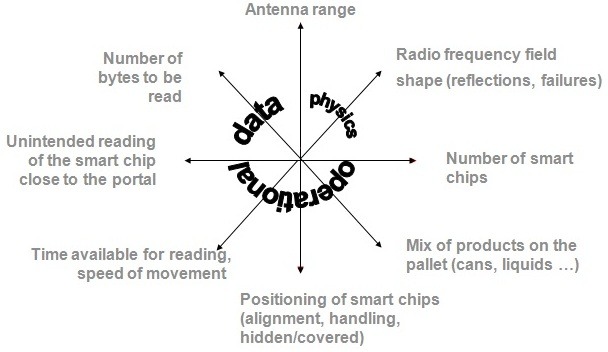


**Tool Box of Modern Technologies**

**Figure:**
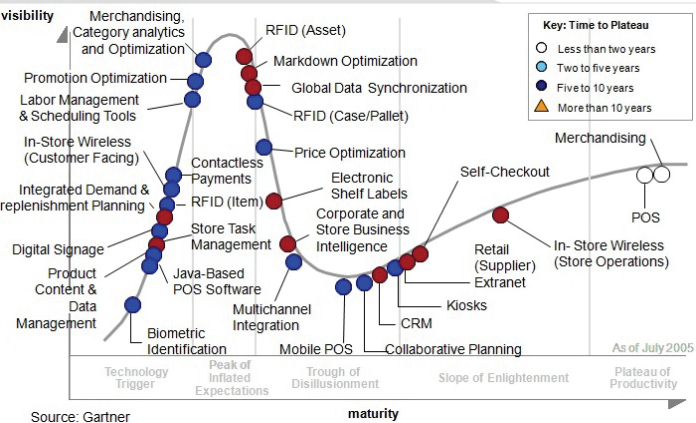


**Personal Shopping Assistant**

**Figure:**
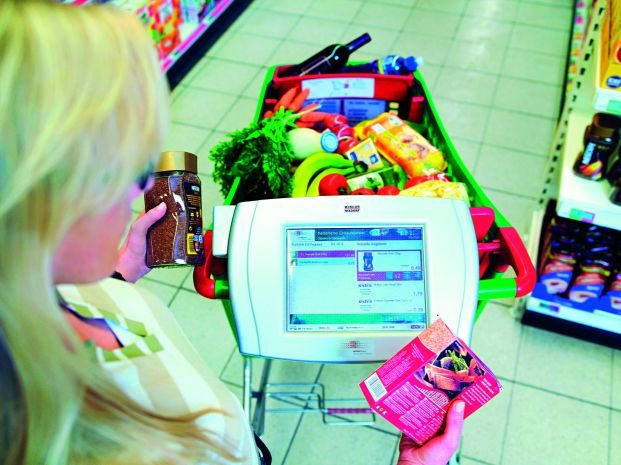


**EAN/GS1**

The European EAN merged with other organizations to become GS1 (Global Standard 1)

**Case Study 2**

In 1994 we had the BSE Scandal (British Cow Disease)

Meat was an anonymous product – nobody knew, if the beef came from the UK!

Meat sales dropped by 25 percent in Germany

Bernd Hallier and six butchers in retail discussed solutions in analogy to EAN!

They created the “EHI-Label” for tracing and tracking of cows and beef

**Sample for Tracement-Label**

**Figure:**
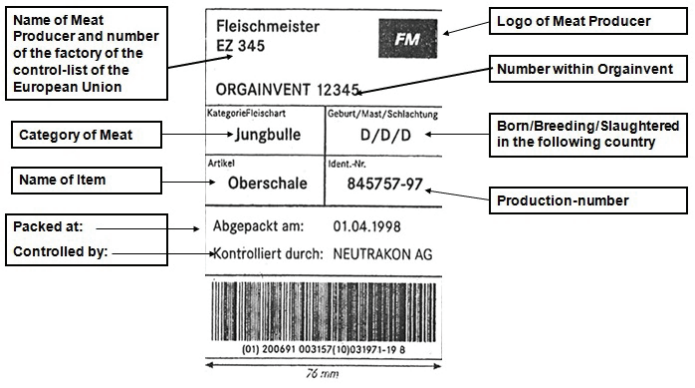


To transform a theoretical approach into applied sciences – suppliers had to join the system.

Cooperating suppliers had been published as benchmarks.

EHI supported the initiatives by publications.

One tool was the monthly magazine Dynamik im Handel.

Another tool had been “special editions” about the topic.

Also two advertisements had been designed and had been placed 18 times in EHI publications in-between 1995 and 2001.

**Figure:**
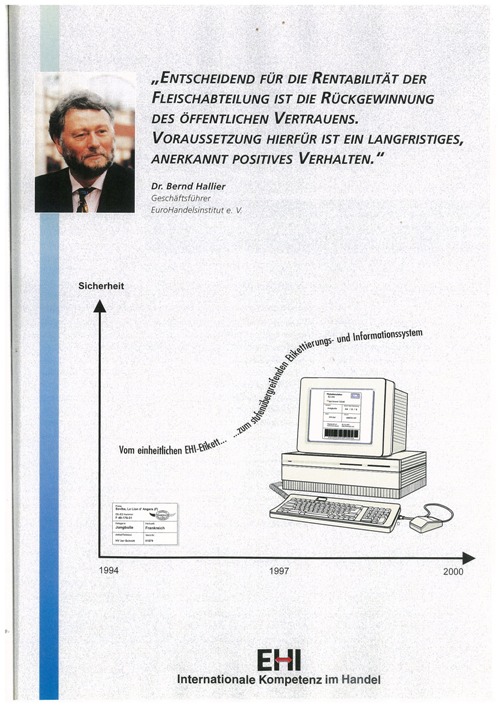


Finally yet importantly each quarter the progress of the EHI-Workshops had been documented. In the year 2000, the pyramid proved 22 steps of effort to regain the consumer trust! 

**Figure:**
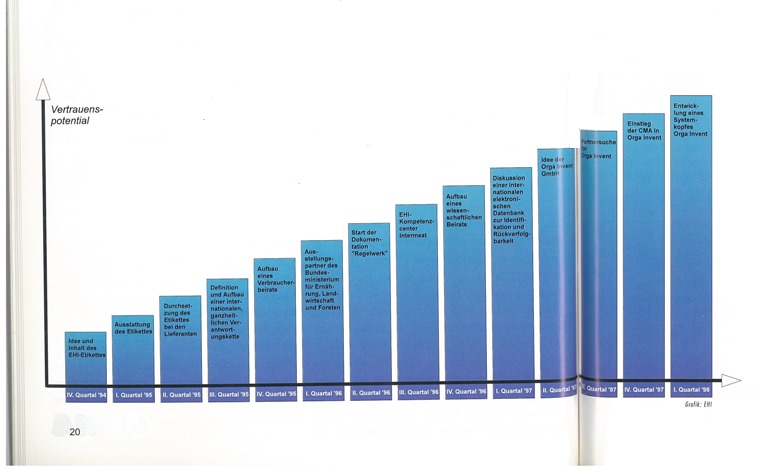


**Total impulses 1995-2000**

**Table:** 

22	43	34	10	9	10
1995	1996	1997	1998	1999	2000

Additionally politicians had been contacted and informed. One of the brochures had been distributed to all members of the German Bundestag. 

The Federal Chancellor Gerhard Schröder had been approached by a letter of Prof.Dr.Hallier on April 7th 2000 to introduce the label compulsory.

On April 18th, the Federal German Minister of Agriculture informed his EU-colleagues about the German decisions

**EHI/Orgainvent**

Meanwhile – pushed by EHI – the Label and the tracing-system became an EU Regulation: 1825/2000 and VO (EG) 999/2001.

While in the end of August the draft version of the new book “From Crisis to Competence” was finished the EU-Commission wrote a draft to change from ear-marks for cows to RFID in 2020!

If – within national boundaries – there will not be enough knowledge institutionalized – then those country’s suppliers will fall out of the globalized distribution/production!

**Case Study 3**

**EHI/GlobalGAP**

Getting involved via Tracking/Tracing into agriculture – EHI started EUREPGAP (European Retailers Produce Good Agricultural Practice) – now **GlobalGAP**

STATISTICS

**Figure:**
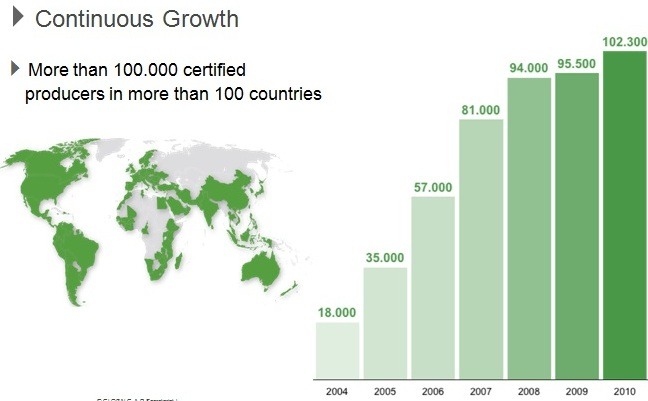


 GLOBALG.A.P GOVERNANCE

**Figure:**
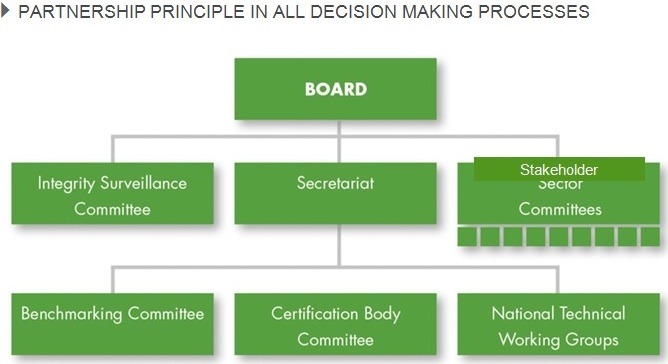


globalg.a.p Simplified

**Figure:**
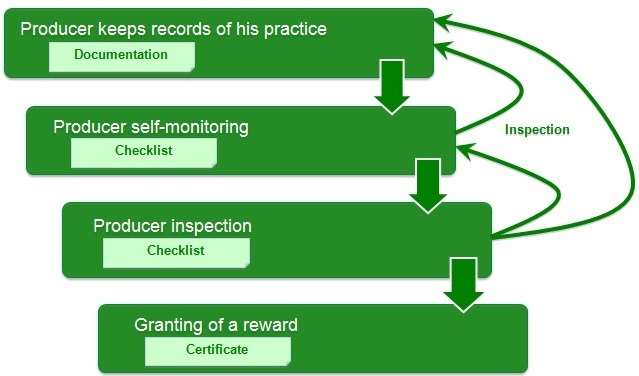


THE FUTURE INTERNET

- Actual developments

**- Farm Management Documentation**

- Documentation of all farm management actions

- Linking from Farm Management Documentation to certification

- Spraying, soil cultivation, field management, ...

- Includes potential information for different issues (e.g. traceability, sustainability)

**- Checklists Documentation**

- Download, fill in and upload checklists to the GLOBALG.A.P Database

- Different checklists for IFA, Add-ons and PFA 

- Information is stored in the database and available for registered users 

- Checklist results can be downloaded as pdf 

**- Open XML Interfaces are available to various existing solutions**

**Conclusion**

Retailers are today’s innovation drivers

The annual turnover of WalMart is as big as the GNP of Switzerland

Taken Hallier’s waves of retail innovation cycles … 

- 7 th: from Mum and Papa-stores to self service

- 8 th: big boxes; shopping centers; backstage; barcoding, scanners, interface to fast moving consumer goods

- 9 th: Internet: B2B/B2C; Chips, RFID interface to agricultural supply (from farm to fork)

- 10 th: M-Aps, M-Shopping, M-payment Individual Health Advice Knowledge Society 

Old advertising:

+ 30 percent price = double size product

New advertising:

+ 30 percent price = healthy food/organics

Corporate Social Responsibility as a sustainability PR/Marketing activity!

